# The Application of Signal Detection Theory to Acceptability Judgments

**DOI:** 10.3389/fpsyg.2020.00073

**Published:** 2020-01-31

**Authors:** Yujing Huang, Fernanda Ferreira

**Affiliations:** Department of Psychology, University of California, Davis, Davis, CA, United States

**Keywords:** signal detection theory, acceptability judgments, d-prime, response bias, one-factor design, two-factor design

## Abstract

Acceptability judgments have been an important tool in language research. By asking a native speaker whether a linguistic token is acceptable, linguists and psycholinguists can collect negative evidence and directly test predictions by linguistic and psycholinguistic theories, which provide important insight into the human language capacity. In this paper, we first give a brief overview of this method including: (1) the linking hypothesis for this method, (2) the controversy about the test, and (3) limitations of the current analysis of the results. Then, we propose a new way of analyzing the data: Signal Detection Theory. Signal Detection Theory has been used in many other psychological research areas such as recognition memory and clinical assessments. In this paper, using two examples, we show how Signal Detection Theory can be applied to judgment data. The benefits of this approach are that it can: (1) show how well participants can differentiate the acceptable sentences from unacceptable ones and (2) describe the participant’s bias in the judgment. We conclude with a discussion of remaining questions and future directions.

## A Brief Overview of Acceptability Judgments

One important type of linguistic data comes from judgments of the well-formedness of linguistic stimuli. An early justification for the use of judgments comes from Chomsky (1957, p. 13), in which it is stated that “[t]he fundamental aim in the linguistic analysis of a language L is to separate the grammatical sequences which are the sentences of L from the ungrammatical sequences.” In this view of language research, grammar is not a set of rules which passively describe what has been seen in a language, but can be viewed as a system for evaluating sequences and making clear predictions regarding what is allowed or disallowed in a language. This makes the linguistic theory falsifiable. Different from methods such as corpus analysis, which can show what structures are possible in a language, linguistic judgments may also reveal what structures are disallowed. These judgments therefore provide negative evidence and allow researchers to directly test predictions regarding what forms a grammar generates and which it does not. Compared to observational data which should not be altered, linguistic judgments can be elicited to target specific hypothesis in a systematic manner.

When judgments were first collected to elicit linguistic intuitions, the procedure was quite informal. These took the form of grammatical judgments. To collect grammatical judgments, linguists would ask their fellow linguist to judge whether a sentence is grammatical or not. Based on this judgment, they would conclude whether a grammatical principle was supported or falsified. The reason the procedure involved querying fellow linguists is because a linguist is tuned to detect subtle grammatical differences and can separate syntactic factors from other influences such as semantics and pragmatics. However, this informal procedure has several potential problems. First, the judgment is based on very limited stimuli which can be as few as one or two token examples (e.g., *Who do you think that left* for the so-called “that-trace” effect; Perlmutter, 1968). Validating a grammatical principle with such a limited sample can be problematic because the generalizability of the judgment across different items is unknown. Second, there may be some implicit bias in the judgment because linguists’ judgments may be unconsciously influenced by the theory they know. Third, it is unclear whether the judgment from a single person can be generalized to the entire population. Fourth, without a standard procedure, the stimuli could be created with different standards by different linguists. Some linguists may compare only minimal pairs. For example, when comparing the well-formedness of prenominal modifiers of different verbs, they may test *the fallen boy* compared to *the jumped boy*, changing only the critical past participle in the sentence. Others may compare *the fallen leaf* with *the laughed boy*. Changing the noun in the phrase could introduce potential confounds. Because of these problems, the reliability of grammatical judgments elicited as described here has been questioned (Schütze, 1996; Edelman and Christiansen, 2003; Wasow and Arnold, 2005; Culicover and Jackendoff, 2010; Gibson and Fedorenko, 2010).

To increase reliability, some researchers advocate using formal procedures that are standardly used in psychology to collect linguistic judgment data (Schütze, 1996; Ferreira, 2005; Culicover and Jackendoff, 2010; Gibson and Fedorenko, 2010; i.a.). In the formal procedure, there need to be multiple items for the same condition with careful controls for potential confounds, and the data are usually collected from several naïve participants who have limited to no exposure to linguistic theory. This formal procedure will increase the sample size of participants and items, will better control for confounds, and avoids bias based on adherence to a particular linguistic theory. While the reliability of the informal procedure has been much debated (Gibson and Fedorenko, 2010; Sprouse and Almeida, 2012; Gibson et al., 2013a,b), it has been shown that acceptability judgments are generally reliable when formal data collection procedures are used that conform to the standards of experimental psychology (Langsford et al., 2018; Linzen and Oseki, 2018). Therefore, in this paper, we restrict our discussion to formal data collection procedures.

However, it is a misnomer to call the data collected using these experimental standards for collecting data as grammaticality judgments. From a theoretical perspective, naïve participants may not be able to separate syntactic factors from other factors such as frequency and plausibility. Their judgments are not based solely on the grammaticality of the stimuli. From a practical perspective, if the participants are asked to judge grammaticality, they are likely to judge the stimuli based on the prescriptive grammar they learned in school rather than providing their intuition about the well-formedness of the stimuli. The better practice may be to ask participants about the acceptability of the stimuli rather than their grammaticality. In asking participants about acceptability, the judgments may be influenced by factors other than the grammaticality of the stimuli, such as frequency, plausibility, pragmatics as well as processing difficulty and processing accuracy. Therefore, it is more appropriate to refer to these judgments as acceptability judgments.

Acceptability judgments differ from grammaticality judgments in an important way: grammaticality reflects the nature of the linguistic stimuli while “acceptability is a percept that arises (spontaneously) in response to linguistic stimuli that closely resemble sentences” (Schütze and Sprouse, 2014). On this view, acceptability is no different from other percepts such as loudness or luminance. One important feature of human perception is that it is never perfect. There is always noise in the perceptual data and in perceptual systems. Indeed, if we ask the same participant to judge different items in the same condition or if we ask different participants to judge the same item, we would not necessarily expect the same response from every participant on every item. If we look at the results from studies that test the reliability of acceptability judgments, we can see that there is indeed between-subject and between-item variability (e.g., Langsford et al., 2018).

This noise can come from many different sources. As we mentioned above, many factors can influence the perception of the acceptability of a sentence, for example, plausibility, frequency, etc. If the event described in a sentence is less plausible, a participant may judge it to be less acceptable although the sentence is perfectly grammatical. Such factors are based on participants’ unique linguistic and nonlinguistic experiences and differ from person to person. They can be controlled as a whole with norming studies but are hard to eliminate for individual participants. As a result, there will be variability in judgments at individual participant and individual item level. In addition, processing difficulty can also influence the acceptability of a linguistic stimulus. For example, a garden-path sentence such as “The horse raced past the barn fell” may be judged as unacceptable although it is not ill-formed. This is because this sentence is hard to parse and the participant may have a hard time building the correct representations of the sentence and therefore will interpret difficulty of processing as evidence for ungrammaticality (Ferreira and Henderson, 1991). Finally, as Gibson et al. (2013a) have argued, input to our language processing mechanisms is not error-free. A participant could provide a judgment based on an input that is not entirely consistent with the stimuli. For example, a participant may misread a sentence because the form of a sentence does not conform to his/her predicted structure and judge an ungrammatical sentence to be grammatical as a result. These are inherent features of our language processing mechanisms and cannot be eliminated either. As none of these sources of noise can be eliminated, there will always be some variance in acceptability judgments.

Another important feature of perception is that there can be some biases in the response. In cases when the stimuli are entirely unacceptable, bias may not be a concern; presumably, nobody will judge a random sequence of words as acceptable, for example. However, in less clear cases, the response bias may have impact on the data. Some participants may be reluctant to judge a sentence as unacceptable and therefore will have a bias to say *yes*. Other participants may tend to be very strict and judge anything that sounds a bit odd to them to be unacceptable (no matter whether it is the form of the sentence, the plausibility of the scenario, or other reasons). These participants have a bias to say *no*. These biases can reduce the difference between theoretically unacceptable and acceptable stimuli and therefore need to be taken into consideration in the data analysis models.

Acceptability judgment data are usually analyzed using some type of significance test, for example, *t*-test (e.g., Clifton et al., 2006; Sprouse, 2011; Sprouse et al., 2013; i.a.) and mixed effect models (e.g., Gibson et al., 2013b; Sprouse et al., 2013, i.a.)^1^. With these tests, a single value of *p* would tell us whether we should reject the null hypothesis and adopt the alternative hypothesis, i.e., these two samples are significantly different from each other. Because these tests compare two samples, some variability is assumed in the data. Therefore, noise is not a problem for these models.

However, these significance tests do not have a built-in mechanism to model response biases. *T*-tests which care about the sample means could be impacted by the bias because the bias may dilute the differences between the two samples. Mixed-effect models can capture the variability at the participant level if a participant random effect is added to the statistical model, but this is still different from modeling response bias^2^. Response biases are not merely random variability across participants. Instead, they are systematic and reflect the criterion a participant sets, i.e., the threshold to judge a stimulus as acceptable. The information of the criterion is overlooked in these significance tests.

In addition to the inability to model biases, there is another factor we need to consider regarding the use of significance tests to evaluate judgment data: How should we interpret any significant results from these models? For example, if the mean of one condition is 0.5 and another is 0.6, given a large sample size, it is likely that a significance test would give a value of *p* that is below our predetermined alpha-level (say, 0.05). Does this significant result mean anything? We could easily run into the standard caveat of significance testing, i.e., the statistical significance may not be meaningful given our theory. One solution to solve this problem is to calculate the effect size. This can be straightforward with the *t*-test but quite complex in mixed-effect models which are more appropriate for tests with multiple subjects and items (Westfall et al., 2014; Brysbaert and Stevens, 2018).

In this section, we gave a brief overview of acceptability judgment in language research. We discussed the linking hypothesis for using acceptability judgments to study language and we also briefly reviewed the nature of judgment data. In the remainder of this paper, we discuss an alternative method of analyzing the acceptability judgment data, i.e., signal detection theory, which models the size of the effect directly and offers a straightforward measure of bias. In the section “Signal Detection Theory and Acceptability Judgments,” we explain SDT and how it can help us better understand the acceptability judgment data. In the sections “Signal Detection Theory and One-Factor-Design Experiments” and “Signal Detection Theory and Two-Factor-Design Experiments,” we provide two examples of the application of SDT to acceptability judgment. And in the final section, “Discussion and Future Directions,” we discuss some remaining questions and future directions.

## Signal Detection Theory and Acceptability Judgments

Signal Detection Theory (SDT) was originally designed to describe the ability of an observer to decide whether the source of a voltage change is noise or signal plus noise (Peterson et al., 1954). Soon afterward, it was adopted by cognitive scientists to measure human decision making in perceptual studies (Tanner and Swets, 1954; Swets et al., 1961). SDT assumes that performance is not perfect and describes how well observers can discriminate or recognize certain signals given the background noise. For example, in recognition memory studies, participants need to decide if a specific stimulus has been presented or not (old or new). There is some ambiguity in this decision, so that given the same stimulus, a participant may judge it as either old or new. SDT captures sensitivity in discriminatory ability so that higher sensitivity means the participant is better able to discriminate old from new items.

SDT has also been adopted in language research by psychologists and linguists to investigate speech perception. In speech perception studies, participants may be asked to categorize sounds according to whether they belong to a certain category or if two sounds are different from each other, corresponding to two commonly used paradigms, “yes-no” and “ABX.” In a study making use of the “yes-no” paradigm, participants decide whether a single signal “A” is present. In the “ABX” paradigm, the two sounds being discriminated (“A” and “B”) are followed by a repetition of one of them, and participants are asked to decide whether “X” is the same as “A” or “B.” Participants’ ability to discriminate the sounds is described by a sensitivity measure. In the design, the stimuli “A” and “B” can be a fixed standard or “roving” on a continuum. Participants’ strategy may change accordingly: With a fixed standard, they may first categorize the stimulus and then compare the categories, and with a “roving” standard, participants may apply a threshold to compare the stimuli and decide if they are different enough to be labeled as such. With different strategies, the calculation of discrimination sensitivity also may differ (Macmillan et al., 1977; Macmillan and Creelman, 2004).

It has been argued that acceptability judgments are a reported perception of acceptability (Chomsky, 1965; Schütze, 1996; Sprouse and Almeida, 2012). In acceptability judgment studies, participants receive a sensory input in the form of a linguistic sequence and are asked to decide whether the sequence is acceptable. This is similar to perceptual studies in other domains, for example, recognition memory studies mentioned above. The SDT was previously adopted by Achimova (2014) to analyze acceptability data related to quantifier scope but the work does not discuss why SDT is appropriate for judgment data, nor does it mention how the different metrics were calculated. In this section, we show why SDT is appropriate for analyzing acceptability judgments and we describe some advantages of using this method as well as different measures in SDT.

**Figure 1:**
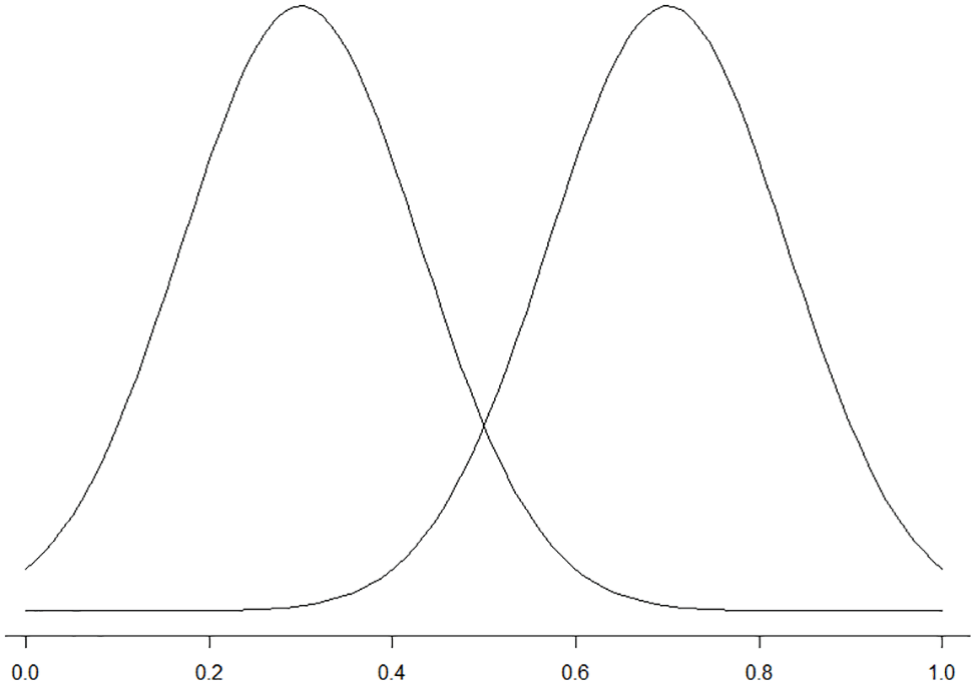
Visual illustration of the probability distributions.

As was discussed in the section “Signal Detection Theory and Acceptability Judgments,” acceptability judgments assume a single underlying construct, i.e., acceptability. Participants need to make a decision regarding this construct: whether a sentence is acceptable or not^3^. For a single category, there is a probability distribution of judgments along the dimension of this construct. As there are two categories of stimuli, acceptable and unacceptable, there are two probability distributions that differ from each other. If we use the x-axis to represent the rating of the items and the height to represent the probability of the rating, we will see two distributions similar to those in Figure 1. Because there is some noise in decision making (participants may not always be able to tell if a sentence is acceptable or not due to various sources of noise), there is an overlapping area in these two distributions. In Figure 1, for example, an item that receives an average rating of 0.2 is likely to be an unacceptable item whereas an item that receives an average rating of 0.8 is likely to be an acceptable item. If an item receives an average rating of 0.5, it is equally likely to be an acceptable or unacceptable item.

Instead of focusing on the distributions of the ratings as significant tests usually do, SDT evaluates the type of decision being made. From the perspective of signal detection theory, in an acceptability judgment experiment, there are two types of stimuli and two possible decisions^4^. This creates four logical combinations. If the stimulus is predicted as acceptable by a linguistic theory and is judged as acceptable, it is a *hit* (i.e., true positive). If the stimulus is predicted as acceptable by a linguistic theory and judged unacceptable, it is a *miss* (i.e., false negatives). If the stimulus is predicted as unacceptable by a linguistic theory but judged as acceptable, it is a *false alarm* (i.e., false positives). If the stimulus is predicted as unacceptable by a linguistic theory and judged as unacceptable, it is a *correct rejection* (i.e., true negative). There are thus two types of correct responses and two types of errors. Table 1 is a summary of these four types of outcomes.

**Table 1:** Categories of judgments based on SDT.

		Signal	
		Acceptable	Unacceptable
**Response**	**Acceptable**	Hit	False alarm
	**Unacceptable**	Miss	Correct rejection

After categorizing the responses, we can calculate the likelihood ratio of each category. For example, the hit rate (H) is the proportion of acceptable trials to which the participant responded “acceptable.” False alarm rate (F) is the proportion of unacceptable trials to which the participant responded “acceptable.” Assuming that *hit* is 20, *false alarm* is 10, *miss* is 5, and *correct rejection* is 15 (see Table 2), hit rate is 20/(20 + 5) = 0.8 and false alarm rate is (10/10 + 15) = 0.4.

$$d’=z\left(H\right)-z\left(F\right)$$

**Table 2:** A toy example of judgment data with number of participant responses in each of the four categories defined by the signal detection analysis.

Hit (20)	False alarm (10)
Miss (5)	Correct rejections (15)

**Figure 2:**
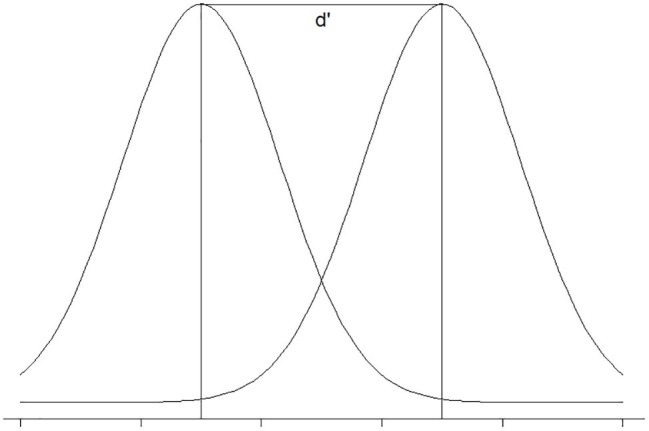
Visual illustration of d’.

The measure of participants’ ability to distinguish between the stimuli (sensitivity, d’) is defined by the inverse of the normal distribution function of H and F (Green and Swets, 1966). In the example above, z(H) is 0.842, z(F) is −0.253, and d’ is z(H) − z(F) which is equal to 1.095. The sensitivity reflects the distance between the acceptable and unacceptable distributions (Figure 2). The larger this number is, the higher the sensitivity (the more distant the two distributions).

In addition to measuring participants’ sensitivity with respect to discriminating the two sets of stimuli, we can also quantify the bias of participants. Bias is caused by participants’ tendency to give one type of response, either “yes” or “no.” As we discussed in the section “Signal Detection Theory and Acceptability Judgments,” if a participant is reluctant to say any sentence is unacceptable, that participant has a “yes” bias; if a participant tends to say any sentence is unacceptable, that participant has a “no” bias. There are many different ways to quantify bias, for example, criterion location (c), relative criterion location (c’), and likelihood ratio (beta). The comparison among these three indices is too technical and beyond the scope of this paper. Here, we use criterion location (c) for illustration purpose. This is because this measure depends monotonically on H and F in the same direction and it is independent of sensitivity d’ (Stanislaw and Todorov, 1999; McNicol, 2005). However, whether it is the best measure of bias for acceptability judgment is an empirical question that needs further investigation.

$$c=-\frac{1}{2}\left(z\left(H\right)+z\left(F\right)\right)$$

**Figure 3:**
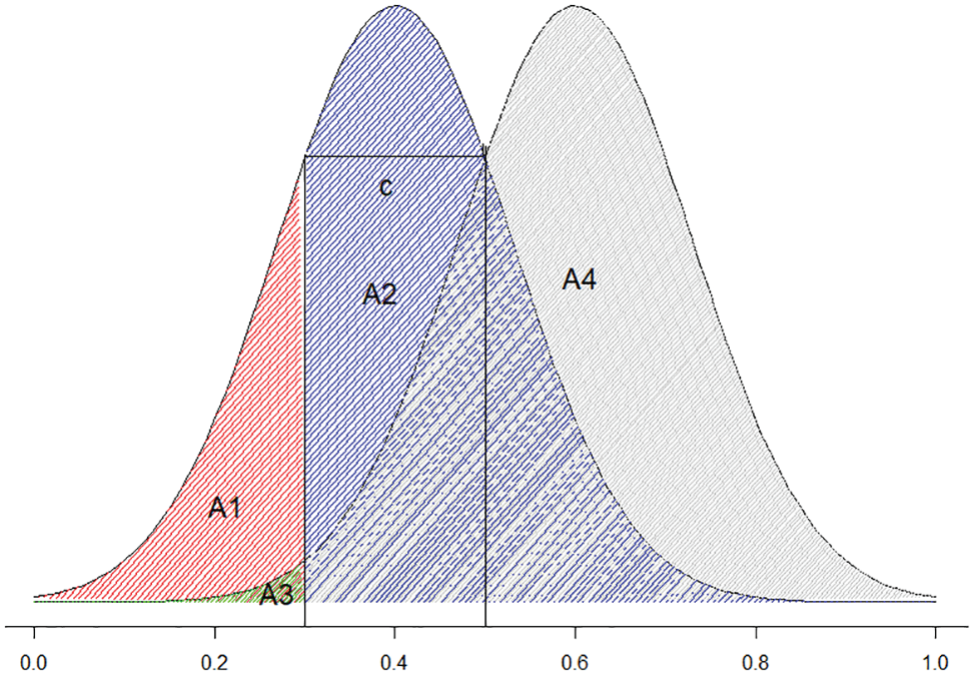
Visual illustration of c.

Criterion location is defined as the negative value of half of the sum of z(H) and z(F). Conceptually, it describes the distance between the selection criterion (the threshold for giving a certain type of response) and the midpoint of the two distributions. When the false alarm and miss rates are equal, c equals 0; when false alarm rate is smaller than misses, c is positive and vice versa. For example, in Figure 3, the threshold is set to 0.2. Any rating higher than 0.2 is judged acceptable and anything lower than 0.2 is judged unacceptable. If the left curve represents unacceptable stimuli and the right curve represents acceptable stimuli, the area A1 (the red shaded area) represents the probability of the correct rejection, A2 (the blue shaded area) represents the probability of the false alarms, A3 (the green shaded area) represents the probability of miss, and A4 (the gray shaded area) represents the probability of hits. In Figure 3, the false alarm area is larger than the misses (A1 > A3), and the bias is negative. This means that the participant has a “yes” bias (is more likely to judge the stimuli as acceptable rather than unacceptable regardless of the properties of the stimuli). In the example of Table 2, c is −0.294. That is a “yes” bias.

SDT is not merely an alternative statistical analysis to acceptability judgment data. It is a different way to think about acceptability judgments. Significance tests assess whether the two samples tested are from the same underlying distribution. This may create an illusion that we are testing the nature of the linguistic stimuli, that is, whether the stimuli are acceptable or not. However, acceptability is not a reflection on the nature of the stimuli. Rather, it reflects how these stimuli are perceived. Therefore, what is tested should not be whether these two sets of stimuli come from the same underlying distribution. Rather, the question should be whether the two sets of stimuli are perceptually differently. SDT is designed to address the latter while significant tests address the former.

## Signal Detection Theory and One-Factor-Design Experiments

In this section, we provide a concrete example of the application of SDT to acceptability judgments with a one-factor design. The data are taken from a study in Huang (2018) ^5^. The aim of the study was to investigate one of the unaccusative diagnostics – the -er nominalization (nominalizing a verb by adding the -er morpheme, e.g., run - > runner). The Unaccusative Hypothesis claims that there are two types of intransitive verbs. The subject of the unergative verb (e.g., *run*) is base-generated as the external argument, whereas the subject of the unergative verb (e.g., *arrive*) is originally generated as the internal argument (Perlmutter, 1978; Maling et al., 1986). Fabb (1984) has argued that -er nominalization only applies to a verb that has an external argument. Therefore, −er nominalizations should be possible for unergative verbs and not unaccusative verbs. Based on the theory, we can construct a study to test if English speakers can distinguish unaccusative verbs and unergative verbs using -er nominalizations. In Huang (2018), each participant was given a list of unaccusative and unergative verbs with the -er nominalization (e.g., *runner* versus *arriver*, where presumably *arriver* seems unacceptable) and was asked to judge if the word was an acceptable English word. For the purposes of this exercise, we use a subset of the data only. In this subset, there were 30 unaccusative verbs and 30 unergative verbs with an -er nominalization. All the items were judged by 20 native English speakers who were naive with respect to the linguistic and psycholinguistic theories. Unaccusativity of the verb was the only factor manipulated in the study, and it had two levels: unaccusative and not unaccusative (i.e., unergative).

### Overall Sensitivity and Bias

To assess whether the unaccusative and unergative conditions are perceived differently, we can calculate the overall sensitivity and bias based on the collective judgments. This means that we ignore individual differences across items and participants. To calculate sensitivity and bias, first we need the frequency of each type of judgment. Those frequencies are given in Table 3.

**Table 3:** Frequency of the choices in each category for the –er nominalization study.

	Unergative	Unaccusative
Acceptable	526	269
Unacceptable	74	331

As we explained above, the unergative condition should be judged as acceptable and therefore, the acceptable responses are *hits* and the unacceptable responses are *misses*. There are 526 *hits* and 74 *misses*. The unaccusative condition should be judged unacceptable and therefore the acceptable responses are *false alarms* and the unacceptable responses are *correct rejections*. There are thus 331 *correct rejections* and 269 *false alarms*. The data are summarized in Table 4.

**Table 4:** Number of participant responses in each of the four categories defined by the signal detection analysis for the -er nominalization study.

Hit (526)	False alarm (269)
Miss (74)	Correct rejections (331)

As shown in the section “Signal Detection Theory and One-Factor-Design Experiments,” hit rate (H) is Hit/(Hit+Miss) which is 526/(526 + 74) = 0.88. False alarm rate (F) is False alarm/(False alarm+Correct rejection) which is 269/(269 + 331) = 0.45. Based on the hit rate and the false alarm rate, we can calculate d’ (sensitivity) and c (bias). The sensitivity d’ is z(H) − z(F) = 1.158 − (−0.130) = 1.288. The bias c is −½(z(H) + z(F)) = −0.5*(1.158–0.130) = −0.514. In the context of the study, the value of d’ is the distance between the unaccusative and unergative distributions, which is 1.288. This is a non-zero value, meaning that participants were able to discriminate unaccusative and unergative stimuli (the perceptual distance between the unaccusative and unergative stimuli is not zero). The negative bias means that the participants (as a whole) have a bias to judge the stimuli as acceptable.

However, before we reach any strong conclusion, we would want to ask if the d’ and bias we estimated from our data reflect the true underlying parameters. Gourevitch and Galanter (1967) provided a way to calculate the variance of d’ and c by using an approximation. The variance of d’ can be calculated by the equation below:

$$var\left(d'\right)=\frac{H\left(1-H\right)}{N_{2}{\left[\Phi \left(H\right)\right]}^{2}}+\frac{F\left(1-F\right)}{N_{1}{\left[\Phi \left(F\right)\right]}^{2}}$$

where N_2_ is the number of signal trials and N_1_ is the number of noise trials. Φ(H) is the height of the normal density function at z(H).

As we have calculated, H is 1.158 and F is −0.130. Based on the equation above, Φ(H) is 0.204 and Φ(F) is 0.396. Var(d’) is 0.00697. The standard error is the square root of the variance: 0.083. The 95% confidence interval is 1.96 standard errors above and below the estimated d’ and therefore is 1.288 ± 1.96*0.083, that is (1.12, 1.45). This means that we can be 95% confident that the true d’ is between 1.12 and 1.45. Critically, this interval does not contain 0. Therefore, the participants were able to discriminate the unaccusative stimuli from the unergative stimuli in the study based on the nominalization test.

The variance of bias is a quarter of the variance of d’ (Macmillan and Creelman, 2004). Therefore, the variance of c is 0.0017, the standard error is 0.042 and the confidence interval is −0.514 ± 1.96*0.042, which is (−0.68, −0.35). This interval is negative and, therefore, there is a bias to judge the stimuli as acceptable.

### Sensitivity and Bias by Participant

In recognition memory research (for an overview of such work, see Rugg and Curran, 2007), sensitivity and bias are usually calculated at each individual participant level. This is because sensitivity and bias describe the perceptions of individual participants and can differ from person to person. Some people may be better at discriminating certain stimuli than others and some people may tend to say “yes” or “no” more than others.

As we discussed in the section “Signal Detection Theory and Acceptability Judgments,” individual linguistic and non-linguistic experiences differ from person to person. Therefore, their judgment of the stimuli can differ from individual to individual. If we want to make a claim about an entire population (e.g., American English speakers), we need to test the hypothesis at the individual level and see if the hypothesis holds across individuals. This is the first step to making any generalization about the population.

The steps to calculate individual sensitivity (d’) and bias (c) are the same as those for the overall d’ and c. Instead of summarizing the data across all participants, we categorize and summarize the responses by each individual. In our example, there were 30 trials in each condition. It is possible that a participant will have perfect accuracy (hit rate equals 1). This would result in an infinite d’. There are two common ways to correct for extreme proportions. One is to add 0.5 to all data cells for that participant. The other is to convert proportion of 0 to 1/(2 N) and 1 to 1–1/(2 N), where N is the number of trials. Here, we choose to add 0.5 to all data cells. This method is proved to be less biased and more conservative (Hautus, 1995).

After calculating the sensitivity and bias for each participant, we can perform inferential statistics on each. Because our question is whether participants can discriminate the two conditions, we want to know if the perceptual distance (d’) is likely to be 0. To answer this question, we can perform a one sample *t*-test to test if 0 is a likely d’ value based on our sample. We found that our sample mean is significantly different from 0 (*t* = 13.19, *p* < 0.001). Therefore, our participants were able to discriminate unaccusative and unergative stimuli.

Following the same logic, we can run a *t*-test and see if the bias is different from 0 (no bias). We find that the bias significantly different from 0 (*t* = −5.73, *p* < 0.001).

## Signal Detection Theory and Two-Factor-Design Experiments

In section Signal Detection Theory and One-Factor-Design Experiments, we gave an example of how SDT can work with one-factor-design experiments. In this section, we show how SDT can be applied to two-factor-design studies. The data in this section are taken from another study in Huang (2018). This study investigated another unaccusative diagnostic: prenominal participles. Prenominal participles refer to the phenomenon where the participle form of a verb can be used as a prenominal modifier of a noun (e.g., *fallen* in *the fallen leaf*). It has been argued that prenominal participles are only possible when the verb is unaccusative and impossible when the verb is unergative (Borer, 1984; Levin and Rappaport, 1986). In Huang (2018), these claims were tested using acceptability judgments^6^. In this study, there were two types of verbs (unaccusative and unergative) and two conditions (control and test). The test condition was a noun phrase with the prenominal modifier (e.g., *the fallen leaf*) and the control condition was a sentence in which the verb was the predicate and the noun was the argument (*The leaf fell.*). Each verb appeared in both the test and control conditions. The control condition was added to ensure that the combination of the verb and the noun was not semantically or pragmatically unacceptable. Two lists of stimuli were created so that each participant only saw the same verb once. The study used a counterbalanced design. The data analyzed in this paper came from 18 participants in each list resulting in a total of 36 participants. There were 30 unaccusative and 30 unergative verbs.

### Overall Sensitivity and Bias

Similar to the previous section, we can calculate the overall sensitivity and bias across all the participants and items. These metrics will tell us whether the participants discriminated unaccusative and unergative stimuli as a whole and whether there is evidence of bias in their responses. Different from the study described in the section “Signal Detection Theory and One-Factor-Design Experiments,” the current study followed a 2 × 2 design. In addition to the verb factor, we added a condition factor where a verb appeared in both the test and control conditions. We do not expect the judgment patterns to be the same in the test and control conditions. In fact, if the prenominal participle test can differentiate unaccusative verbs from unergative verbs, we would expect participants to discriminate the two types of verbs in the test condition but not in the control condition (because the control condition does not have prenominal modifiers and is therefore acceptable for both verb types). Thus, we need to analyze these two conditions separately.

For the test condition, the number of acceptable and unacceptable judgments for the two verb types is summarized in Table 5.

**Table 5:** Frequency of the choices in the test condition for the prenominal participle study.

	Unaccusative	Unergative
Acceptable	285	118
Unacceptable	255	422

As we explained above, the unaccusative condition should be judged as acceptable and therefore the acceptable responses are *hits* and the unacceptable responses are *misses*. There are 285 *hits* and 255 *misses*. The unergative condition should be judged unacceptable and therefore the acceptable responses are *false alarms* and the unacceptable responses are *correct rejections*. There are 118 *false alarms* and 422 *correct rejections*. The data are summarized in Table 6.

**Table 6:** Number of participant responses in each of the four categories defined by the signal detection analysis for the prenominal participle study.

Hit (285)	False alarm (118)
Miss (255)	Correct rejections (422)

Based on Table 6, d’ for the test condition is 0.847 and c is 0.354. In the context of the study, the value of d’ is the distance between the unaccusative and unergative distributions, which is 0.847. This is a non-zero value, meaning that the participants can discriminate unaccusative and unergative stimuli (the perceptual distance between the unaccusative and unergative stimuli is not zero). The positive bias means that the participants (as a whole) have a bias to judge the stimuli as unacceptable.

As in the section “Signal Detection Theory and Two-Factor-Design Experiments,” we can calculate the standard error and 95% confidence interval of d’. The standard error is 0.0809 and the confidence interval is (0.69, 1.01). This interval does not contain zero which means that there is a non-zero perceptual distance between unaccusative and unergative stimuli. In other words, the participants were able to discriminate these two sets of stimuli.

Following the same steps, we can also calculate d’ and c in the control condition. Table 7 summarizes the frequency of responses.

**Table 7:** Frequency of the choices in the control condition for the prenominal participle study.

	Unaccusative	Unergative
Acceptable	507	516
Unacceptable	33	24

One thing to note is that the categorization of the control condition is artificial, because all control sentences should be judged as acceptable no matter what type of verb they include. However, when we analyze the data, we need to categorize the responses in the same way as in the test condition so that the interpretation of d’ and c remains the same and can be compared across test and control conditions. If an unaccusative stimulus is judged as acceptable, it is a *hit* and otherwise it is a *miss*. There are 285 *hits* and 255 *misses*. Likewise, if an unergative stimulus is judged as unacceptable, it is a *correct rejection*, and otherwise it is a *false alarm*. There are 422 *correct rejections* and 118 *false alarms*. The data are summarized in Table 8. In hypothesis tests such as the *t*-test, we assume that the null hypothesis is true and test if we should reject this assumption. Here, we assume that the two distributions of interest can be discriminated (the unaccusative stimuli should be acceptable and unergative stimuli should be unacceptable) and test whether this is true.

**Table 8:** Number of participant responses in each of the four categories defined by the signal detection analysis for the control condition of the prenominal participle study.

Hit (507)	False alarm (516)
Miss (33)	Correct rejections (24)

Based on Table 8, the control condition has a d’ of −0.156 and a c of −1.623. The standard error of d’ is 0.127 and the 95% confidence interval is (−0.41, 0.09). This confidence interval contains 0. Therefore, we have no evidence that the participants discriminated the unaccusative and unergative stimuli in the control condition. This is consistent with our expectations, since the verb+noun sequence was predicted to be acceptable for both verb types. There is no theoretical reason why these two sets of stimuli would differ in the control condition.

Taken together, the results show that participants were able to discriminate unaccusative and unergative verbs in the prenominal participle form, and this ability is not confounded with any semantic and pragmatic differences, since the verbs were not distinguished in the control condition. The calculation of confidence interval for c is the same as that in the one-factor design section and so we will not repeat it here.

### Sensitivity and Bias by Participant

The calculations of sensitivity and bias by participant are very similar to those of the section Signal Detection Theory and One-Factor-Design Experiments. The only difference is that we need to treat the test and control conditions separately, as we did in the section “Overall Sensitivity and Bias.” The detailed calculation is available in supplemental R code and so we will not repeat the calculations here. After the calculation, we have two sets of d’ values for each participant: a set of d’ values for the test condition and a set of d’ values for the control condition. We perform a paired *t*-test to compare these two sets of d’ values. This comparison tells us whether our participants’ ability to discriminate the unaccusative and unergative stimuli is different in the test condition and the control condition. We found a significant difference between the test and control conditions (*t* = 9.30, *p* < 0.001). Therefore, our participants differentially discriminated these two types of verbs in these two conditions.

### Sensitivity and Bias by Item

It has been argued that, in psycholinguistic research, items should not be treated as a fixed effect (Clark, 1973). It is important to know if the effect we find is driven by certain items or it is true across the board, and therefore it is generally accepted that items should be included as random effects in our statistical models. In this section, we show how to calculate sensitivity and bias in by-items analyses.

In the prenominal participle study, each verb/item appeared in two different conditions: test and control. Each item therefore is associated with four types of responses, as shown in Table 9. Here, we want to compare if the response for the test condition is different from that for the control condition. We use the control condition as the baseline because all items in this condition should be acceptable. Therefore an acceptable response in the control condition is a *hit* and an unacceptable response is a *miss*. We assume that an acceptable response in the test condition is a *false alarm* and unacceptable response is a *correct rejection*. With this categorization, if the d’ ends up being zero, we know that there is no difference (perceptual distance) between our test and control conditions.

**Table 9:** Categorization of judgment data for the prenominal participle study by item.

	Control	Test
Acceptable	Hit	False alarm
Unacceptable	Miss	Correct reject

With the above categorization, we can make a frequency table for each item and calculate a d’ and a c value for each item. The d’ value indicates how different the test condition of the item is from the control condition. The c value indicates if the participants show any response bias for this item.

After calculating the d’ for each item, we can assess whether the values for d’ in the unaccusative condition are different from those in the unergative condition using a *t*-test. We find a significant difference (*t* = −4.37, *p* < 0.005). However, here we need to be careful with the interpretation of the results. We find that the average d’ is larger for the unergative than for the unaccusative condition. Because the d’ in our calculation is the perceptual distance between the test condition and the control condition (acceptable condition), the larger this number is, the more different the test condition is from the acceptable condition (less acceptable). Therefore, a larger d’ means that the unergative condition is less acceptable. In our example, the larger average d’ in the unergative condition means that the unergative condition is less acceptable than the unaccusative condition.

## Discussion and Future Directions

In this paper, we first discussed why acceptability judgments can be a useful tool for language research, and we also considered the reliability of the method. Then, we showed how SDT can be applied to analyze the judgment data. After introducing some fundamental concepts, we showed how sensitivity and bias are calculated and how they can help us better interpret acceptability judgment data. In this section, we discuss the assumptions behind the models used in the sections “Signal Detection Theory and One-Factor-Design Experiments” and “Signal Detection Theory and Two-Factor-Design Experiments” and some future directions of research.

The models presented in the sections “Signal Detection Theory and One-Factor-Design Experiments” and “Signal Detection Theory and Two-Factor-Design Experiments” embody two important assumptions: (1) the data follow a Gaussian distribution and (2) the variances of the two distributions are equal. These assumptions are also made by many significant tests such as *t*-test and ANOVA. If the variances are unequal, a single signal detection study will not be sufficient to determine sensitivity and bias. Instead, we will need to have several conditions varying in bias or we will have to conduct a rating-scale experiment (Wickens, 2002; McNicol, 2005). Due to the complexity of this issue, we do not discuss the unequal variance model in this paper. Researchers who are interested in this topic should consult Wickens (2002) and McNicol (2005), among others.

There are some additional interesting questions that can be addressed using SDT. First, it can help us quantify the discriminability of different conditions. Imagine we have three groups of stimuli, Group A (the baseline acceptable control), Group B, and Group C, with stimuli in the two groups differing in their average degree of acceptability. We can calculate a d’ using Group A and B which gives us the perceptual distance between Group A and B. We can also calculate a d’ using Group A and C which gives us the perceptual distance between Group A and C. Assuming that the d’_A_B_ is 1.2 and d’_A_C_ is 2.2, we can tell that Group B has less perceptual distant from the acceptable condition than Group C (Group B is more acceptable). Although the judgment is binary, d’ as a continuous metric can give us a continuous measure of the perceptual distance between different stimuli across a continuum.

We can also compare performance in different populations, which is a more canonical way of using SDT. For example, we can give non-native speakers and native speakers the same stimuli and then compare their performance (d’). If the d’ of the native speakers is larger than that of the non-native speaker (as we would expect), we know that native speakers can discriminate the stimuli more accurately, that is, their sensitivity for the phenomenon being tested is better.

There are many remaining questions that need more investigation. In the section “Signal Detection Theory and One-Factor-Design Experiments,” we presented one possible measure of bias. We chose this measure to illustrate how bias can be interpreted in the context of acceptability judgments. As we mentioned, there are some alternative measures of bias. Which one best describes the bias in the acceptability judgment data is an empirical question that needs further investigation.

In the paper, we limited our discussion to binary judgments because research has shown that the results for acceptability judgments tend to be consistent regardless of whether the scale provides more than two response categories (Bader and Häussler, 2010). However, we can use SDT for rating judgments involving a non-binary scale as well. One thing to note is that, for acceptability judgments, we usually give participants a scale and ask them to rate the acceptability of the stimuli on that scale. In the context of SDT, rating judgments are performed differently. What participants rate on the scale is not the acceptability of the stimuli but rather how confident they are in their judgment. They still need to make a binary judgment on the acceptability of the stimuli. In addition to that, they need to indicate their confidence level on a scale. One question we can ask is to what degree the acceptability rating and the confidence rating are correlated. Acceptability is believed to be continuous and the gradient judgments from acceptability ratings are believed to reflect the continuous nature of acceptability. However, there is another possibility: the gradient data are created by another factor that is orthogonal to an item’s acceptability. One candidate for such an orthogonal factor is confidence level associated with the judgments. By testing the correlation between the acceptability rating and the confidence rating, we can tease apart these two possibilities. If these two factors are uncorrelated, we can exclude the possibility that the gradient judgment is caused by variation in participants’ levels of confidence. However, if these two factors correlate significantly, then the gradient data pattern is likely to be caused by participants’ confidence level rather than the commonly believed acceptability continuum. In this case, we may need to consider an alternative interpretation of the gradient judgments. It is possible that acceptability is a not a real continuous measure, but the results of these tests are confounded with subjects’ confidence about their responses, which is continuous.

SDT can help us address some important questions, including how participants’ perceptions of acceptability vary when the linguistic properties of the stimuli are changed in theoretically interesting ways. For example, it is possible to test whether the effect of grammatical violation on acceptability is cumulative. If the effect is cumulative, we would expect stimuli that violate more rules to be judged less acceptable than stimuli that violate fewer rules. For example, if a set of stimuli violates agreement principles of the grammar whereas another set violates both agreement and case features, the second set should be judged less acceptable than the first set, and this difference should be reflected in their d’s. If the ratings of the stimuli can correctly reflect the difference in the degree of acceptability of these stimuli, we expect the d’s in these two conditions to differ. We can also change other factors of the stimuli such as the plausibility of the scenario described by the stimuli. This is likely to change participants’ judgments: For example, they may judge the more plausible stimuli to be more acceptable. This should happen for both unacceptable and acceptable stimuli. If plausibility and acceptability operate independently, the perceptual distance (d’) between these two sets of stimuli should not change because it reflects the acceptability differences between the stimuli. The bias should change because the participants are biased to judge all stimuli to be acceptable. By manipulating different factors in the experiment and seeing how d’ and c changes, we can have a better understanding on how plausibility interacts with acceptability. Overall, we believe this approach making use of SDT to analyze binary acceptability responses has the potential to expand our understanding of what such judgments reflect and will allow us to continue to refine our theories of linguistic representation and processing.

## Data Availability Statement

The datasets generated for this study can be found in the [Open Science Framework] [https://osf.io/pdcye/].

## Author Contributions

YH and FF conceived of the presented idea, discussed the results, and contributed to the final manuscript. YH developed the theory and performed the computations. FF verified the analytical methods.

### Conflict of Interest

The authors declare that the research was conducted in the absence of any commercial or financial relationships that could be construed as a potential conflict of interest.

## Footnotes

^1^An anonymous reviewer pointed out that in the discussion of statistical methods, one method that is worth mentioning is Bayesian statistics. Bayesian statistics provides a probability distribution over hypotheses. It can be especially useful when we want to integrate prior beliefs into the analysis. However, it shares some limitation with frequentist tests when modeling perpetual data (e.g., it does not have an explicit way to quantify bias).

^2^An anonymous reviewer pointed out that a random intercept can provide some insights on bias by showing that “the acceptability judgment value never goes below a certain threshold for a given subject.” However, there are two limitations with this random intercept argument. First, the inference concerning bias is indirect (we need to compare the intercept with some value that must be separately calculated). Second, when the subject effect is treated as a random effect, it is essentially seen as variance that researchers do not care about (compared to a main effect). However, bias is not random noise: as we discuss in this paragraph, bias reflects the decision criterion of a participant.

^3^An anonymous reviewer has pointed out that acceptability is gradient rather than binary. To clarify, when we talk about binary decisions, we refer to the nature of the task (i.e., in the judgment study, the participants are asked to judge if a stimulus is acceptable). This does not require the underlying construct to be binary. To make binary judgments on a continuous underlying construct, the participant must decide on a threshold beyond which all the stimuli are acceptable and below which all the stimuli are unacceptable. This is how a continuous underlying construct can be measured with a binary decision. This follows the same logic as tasks in memory research in which the participant judges the familiarity of the stimuli (continuous) by providing binary judgments (if the stimuli have been seen before).

^4^Bader and Häussler (2010) show that gradient judgment data and binary judgment data are highly correlated. Therefore, in this paper, we adopt the binary task which makes the data structure simple and straightforward. An anonymous reviewer pointed out that “there are self-evident judgments, whose replication/correlation across different elicitation techniques is unsurprising, and then there are potentially questionable judgments, which may introduce some variation across techniques/samples”. In Bader and Häussler (2010), many different phenomena were tested and in their results, it is clear that the judgments are not polarized (which is what we would expect if the sentences are cleared acceptable or unacceptable). Therefore, Bader and Häussler (2010) did not only test self-evident judgments.

^5^The description in this section is an oversimplification of the actual study. The original data were based on a 7-point scale. We split the data into binary choices at the midpoint (any score below 4 is transformed to 0 and the rest is 1). We only took a subset of the data for illustration purpose. We do not intend to make any theoretical conclusion.

^6^This is again an oversimplification of the study. The counterbalanced structure was also altered to work with SDT. We do not intend to make any theoretical conclusion with this example. All interpretations of the data are for illustration purposes to show what d’ and c mean in a real dataset.

## References

[ref1] Achimova A. (2014). Resolving wh−/quantifier ambiguities: Integrating theoretical and experimental perspectives. Doctoral dissertation. Rutgers University-Graduate School-New Brunswick.

[ref2] Bader M. Häussler J. (2010). Toward a model of grammaticality judgments. J. Linguist. 46, 273–330. 10.1017/S0022226709990260

[ref3] Borer H. (1984). “The projection principle and rules of morphology” in Proceedings of the Fourteenth Annual Meeting of NELS. eds. Jones C. Sells P. (Amherst: GLSA, University of Massachusetts), 16–33.

[ref4] Brysbaert M. Stevens M. (2018). Power analysis and effect size in mixed effects models: a tutorial. J. Cogn. 1, 1–20. 10.5334/joc.10 PMC6646942 31517183

[ref6] Chomsky N. (1957). Syntactic structures. The Hague: Mouton.

[ref501] Chomsky N. (1965). Aspects of the theory of syntax. Cambridge, MA: MIT Press.

[ref7] Clark H. H. (1973). The language-as-fixed-effect fallacy: a critique of language statistics in psychological research. J. Verbal Learn. Verbal Behav. 12, 335–359. 10.1016/S0022-5371(73)80014-3

[ref8] Clifton C. Jr. Fanselow G. Frazier L. (2006). Amnestying superiority violations: processing multiple questions. Linguist. Inquiry 37, 51–68. 10.1162/002438906775321139, PMID: 17356682 PMC1820880

[ref9] Culicover P. W. Jackendoff R. (2010). Quantitative methods alone are not enough: response to Gibson and Fedorenko. Trends Cogn. Sci. 14, 234–235. 10.1016/j.tics.2010.03.012

[ref500] Edelman S. Christiansen M. H. (2003). How seriously should we take minimalist syntax? A comment on Lasnik. Trends Cogn. Sci. 7, 60–61. 10.1016/s1364-6613(02)00045-1 12584020

[ref10] Fabb N. A. J. (1984). Syntactic affixation. Doctoral dissertation. Cambridge (MA): Massachusetts Institute of Technology.

[ref11] Ferreira F. (2005). Psycholinguistics, formal grammars, and cognitive science. The Linguist. Rev. 22, 365–380. 10.1515/tlir.2005.22.2-4.365

[ref12] Ferreira F. Henderson J. M. (1991). Recovery from misanalyses of garden-path sentences. J. Mem. Lang. 30, 725–745. 10.1016/0749-596X(91)90034-H

[ref13] Gibson E. Bergen L. Piantadosi S. T. (2013a). Rational integration of noisy evidence and prior semantic expectations in sentence interpretation. Proc. Natl. Acad. Sci. USA 110, 8051–8056. 10.1073/pnas.1216438110 23637344 PMC3657782

[ref14] Gibson E. Fedorenko E. (2010). Weak quantitative standards in linguistics research. Trends Cogn. Sci. 14, 233–234. 10.1016/j.tics.2010.03.005, PMID: 20363175

[ref15] Gibson E. Piantadosi S. T. Fedorenko E. (2013b). Quantitative methods in syntax/semantics research: a response to Sprouse and Almeida. Lang. Cogn. Process. 28, 229–240. 10.1080/01690965.2012.704385

[ref16] Gourevitch V. Galanter E. (1967). A significance test for one parameter isosensitivity functions. Psychometrika 32, 25–33. 10.1007/BF02289402, PMID: 5232570

[ref17] Green D. M. Swets J. A. (1966). Signal detection theory and psychophysics. Vol. 1. New York: Wiley.

[ref18] Hautus M. J. (1995). Corrections for extreme proportions and their biasing effects on estimated values of d′. Behav. Res. Methods Instrum. Comput. 27, 46–51. 10.3758/BF03203619

[ref19] Huang Y. (2018). Linking form to meaning: Reevaluating the evidence for the unaccusative hypothesis. Doctoral dissertation. Available at: http://nrs.harvard.edu/urn-3:HUL.InstRepos:40049976

[ref20] Langsford S. Perfors A. Hendrickson A. T. Kennedy L. A. Navarro D. J. (2018). Quantifying sentence acceptability measures: reliability, bias, and variability. Glossa: J. Gen. Linguist. 3, 1–37. 10.5334/gjgl.396

[ref21] Levin B. Rappaport M. (1986). The formation of adjectival passives. Linguist. Inquiry 17, 623–661.

[ref22] Linzen T. Oseki Y. (2018). The reliability of acceptability judgments across languages. Glossa: J. Gen. Linguist. 3:100. 10.5334/gjgl.528

[ref23] Macmillan N. Creelman C. (2004). Detection theory: A user's guide. New York: Psychology Press.

[ref24] Macmillan N. A. Kaplan H. L. Creelman C. D. (1977). The psychophysics of categorical perception. Psychol. Rev. 84, 452–471. 10.1037/0033-295X.84.5.452, PMID: 905471

[ref5] Maling J. Rizzi L. Burzio L. (1986). Italian syntax: A government-binding approach. Vol. 1. Dordrecht: Springer Netherlands.

[ref25] McNicol D. (2005). A primer of signal detection theory. New York: Psychology Press.

[ref26] Perlmutter D. M. (1968). Deep and surface structure constraints in syntax. Doctoral dissertation. MIT.

[ref27] Perlmutter D. M. (1978). “Impersonal passives and the unaccusative hypothesis” in Proceedings of the annual meeting of the Berkeley linguistics society. Vol. 4 (Berkeley: University of California), 157–190. Available at: https://escholarship.org/uc/item/73h0s91v

[ref28] Peterson W. W. T. G. Birdsall T. Fox W. (1954). The theory of signal detectability. Trans. IRE Prof. Group Inf. Theory 4, 171–212. 10.1109/TIT.1954.1057460

[ref29] Rugg M. D. Curran T. (2007). Event-related potentials and recognition memory. Trends Cogn. Sci. 11, 251–257. 10.1016/j.tics.2007.04.004, PMID: 17481940

[ref30] Schütze C. T. (1996). The empirical base of linguistics: Grammaticality judgments and linguistic methodology. Chicago: University of Chicago Press.

[ref31] Schütze C. Sprouse J. (2014). “Judgment data” in Research Methods in Linguistics. eds. Podesva R. Sharma D. (Cambridge: Cambridge University Press), 27–50.

[ref32] Sprouse J. (2011). A validation of Amazon mechanical Turk for the collection of acceptability judgments in linguistic theory. Behav. Res. Methods 43, 155–167. 10.3758/s13428-010-0039-7, PMID: 21287108 PMC3048456

[ref33] Sprouse J. Almeida D. (2012). Assessing the reliability of textbook data in syntax: Adger’s Core syntax. J. Linguist. 48, 609–652. 10.1017/S0022226712000011

[ref34] Sprouse J. Schütze C. T. Almeida D. (2013). A comparison of informal and formal acceptability judgments using a random sample from linguistic inquiry 2001–2010. Lingua 134, 219–248. 10.1016/j.lingua.2013.07.002

[ref35] Stanislaw H. Todorov N. (1999). Calculation of signal detection theory measures. Behav. Res. Methods Instrum. Comput. 31, 137–149. 10.3758/BF03207704, PMID: 10495845

[ref36] Swets J. A. Tanner W. P. Jr. Birdsall T. G. (1961). Decision processes in perception. Psychol. Rev. 68, 301–340. 10.1037/h0040547, PMID: 13774292

[ref37] Tanner W. P. Jr. Swets J. A. (1954). A decision-making theory of visual detection. Psychol. Rev. 61, 401–409. 10.1037/h0058700, PMID: 13215690

[ref38] Wasow T. Arnold J. (2005). Intuitions in linguistic argumentation. Lingua 115, 1481–1496. 10.1016/j.lingua.2004.07.001

[ref39] Westfall J. Kenny D. A. Judd C. M. (2014). Statistical power and optimal design in experiments in which samples of participants respond to samples of stimuli. J. Exp. Psychol. Gen. 143, 2020–2045. 10.1037/xge0000014, PMID: 25111580

[ref40] Wickens T. D. (2002). Elementary signal detection theory. USA: Oxford University Press.

